# Call to Action for Improving Community Health While Reducing Health Disparities

**DOI:** 10.31486/toj.23.0040

**Published:** 2023

**Authors:** Eboni G. Price-Haywood

**Affiliations:** ^1^ Ochsner-Xavier Institute for Health Equity and Research, New Orleans, LA; ^2^ Academics Division/Research, Ochsner Clinic Foundation, New Orleans, LA; ^3^ The University of Queensland Medical School, Ochsner Clinical School, New Orleans, LA

The coronavirus disease 2019 (COVID-19) pandemic highlighted for the nation the longstanding disproportionate impact of health disparities on socially disadvantaged populations. In Louisiana, racial disparities in hospitalization, in-hospital death, and community and workplace seroprevalence of COVID-19 were documented during the early phase of the pandemic.^1-3^ These disparities were associated with underlying racial differences in rates of comorbid chronic conditions combined with differences in risk for viral exposure and delayed receipt of care. Notwithstanding, the Louisiana health score reports have demonstrated for years disparities in health among select subpopulations.^4^ Health disparities, as defined by Healthy People 2030, are “health differences that are closely linked with economic, social, or environmental disadvantage.”^5^ Adversely affected populations have systematically experienced barriers to health based on race/ethnicity, religion, socioeconomic status, age, sex, gender identity, sexual orientation, mental health, disabilities (physical/cognitive), geographic location (rural/urban), and other characteristics historically linked to discrimination.

Within this context, America's Health Rankings (AHR), the longest running state-by-state health analysis, has consistently ranked Louisiana as one of the unhealthiest states in the United States.^6^ The rank scoring system is based on analyses of publicly available datasets covering more than 340 measures. Ranking measures are selected based on representation of issues that affect population health, availability of data at the state level, and accessibility of common measures across all 50 states, and the selected measures must be current and updated periodically. The 2022 Annual Health Report includes 83 measures from 29 data sources. Key measures are grouped into the following categories, each of which has a different weight contributing to the final ranking: health behaviors (0.2), physical environment (0.1), social and economic factors (0.3), clinical care (0.15), and health outcome (0.25). The sum of weighted scores generates state rankings of 1 (healthiest) to 50 (least healthy).

In 2022, AHR once again ranked Louisiana as 50th.^7^ Economic hardship was ranked as the most impactful factor for this low rating. The economic hardship index compares economic conditions between communities and is a composite score of 6 measures: unemployment, dependency, education, per capita income, crowded housing, and poverty.^8^ The rate of unemployment has community-wide impacts related to financial stress. Dependency, defined as the proportion of the population under age 18 or over 64 years, has implications for potential hardships related to caring for dependents. Education, the proportion of the population over age 25 years without a high school degree, has implications for local job opportunity and potential income. Per capita income represents the relative wealth of a community. Crowded housing, the proportion of housing units with more than 1 person per room, can adversely impact personal well-being. Finally, living below the federal poverty level is linked to poor health outcomes.^9^ Overall, a higher score for economic hardship is associated with lower life expectancy.

The relative impact of economic hardship on Louisiana's health ranking highlights the importance of social determinants of health (SDoH). The World Health Organization (WHO) defines SDoH as “the conditions in which people are born, grow, live, work, and age.”^10^ SDoH influence 80% of health outcomes, while clinical care only influences 20%.^11^ As exemplified by the WHO conceptual framework for addressing SDoH,^10^ the solution for abating the negative impact of SDoH on health equity lies upstream of the health system. Individuals are born into socioeconomic and political contexts which create social structures that can generate an unequal distribution of factors impacting their exposure and vulnerability to health-compromising conditions (living/working conditions, food availability, behavioral/biological/psychosocial factors). Health systems are downstream of this complex array of social determinants. Therefore, health care providers can either mitigate or worsen health disparities.

As such, in 2022, a number of national agencies released strategies for incentivizing integration of health equity in health care delivery models. The Centers for Medicare & Medicaid Services (CMS) released the CMS Framework for Health Equity 2022-2023^12^ and introduced the Accountable Care Organization Realizing Equity, Access, and Community Health (REACH) Model.^13^ Moreover, the Joint Commission now requires that organizations establish health equity leaders, standardize structures and processes to detect and address health care disparities, and fully integrate these efforts with existing quality improvement activities within the organization as with other priority issues. For accountability and transparency, *U.S. News and World Report* introduced new health equity measures to its public ratings.^14^ Similarly, the Leapfrog Group survey has introduced health equity questions but is not yet publicly reporting scores.^15^ All of these initiatives are rapidly driving organizational changes for which health care systems must be prepared.

In advance of these initiatives, in November 2020, Ochsner Health, Louisiana's largest integrated delivery health system, announced a 10-year commitment to improving the overall health of Louisiana by increasing the state ranking from 50th to 40th by 2030.^16^ Given that most factors impacting health are upstream of the health system, Ochsner leadership recognized the quintessential importance of functioning as a catalyst for change by engaging stakeholders across different industries (education/workforce/job/economic development, health care, public health, health policy). The Ochsner Healthy State initiative is a call to action for diverse organizations to work together within the scope of their respective industries to execute a shared mission. Current priority areas are strategically linked to AHR key drivers of Louisiana's low health rankings (education and workforce, cancer and chronic conditions, smoking cessation, broadband access, food insecurity, obesity).

Concurrent with the Healthy State initiative, Ochsner committed to improving health equity among the populations it serves. To guide this scope of work, the health system collaborated with Xavier University of Louisiana to form the Ochsner-Xavier Institute for Health Equity and Research (OXIHER).^17^ The OXIHER mission is to improve the overall health of all our communities, reduce health inequities, develop innovative health care delivery models, and model equitable and respectful care. The OXIHER team is employing outcomes research, population health, workforce development and education initiatives, community engagement, and health policy to investigate and create solutions for the challenges we face in Louisiana.

Now is the time to take action to change the health of our communities. As a society, we are long overdue for implementing solution-oriented approaches to improving health equity. National awareness of the critical need to close longstanding gaps was largely driven by the preponderance of health outcome data observed during a global crisis. The COVID-19 pandemic serendipitously granted the nation an opportunity to revamp how we conceptualize health and well-being, understand the role of social determinants, and develop data-driven innovative models to improve community health.
